# Percutaneous Mitral Valve Repair in Patients with Severe Mitral Regurgitation and Acute Decompensated Heart Failure

**DOI:** 10.3390/jcm10245849

**Published:** 2021-12-13

**Authors:** Anna Turyan Medvedovsky, Dan Haberman, Mahsati Ibrahimli, Ivaylo Tonchev, Yonatan Rashi, Alona Peretz, Sara Shimoni, Ortal Tuvali, Haim Danenberg, Ronen Beeri, Mony Shuvy

**Affiliations:** 1 The Jesselson Integrated Heart Center, Shaare Zedek Medical Center, Shmu’el Bait St. 12, Jerusalem 9103102, Israel; drannia2004@gmail.com (A.T.M.); yrashi1@gmail.com (Y.R.); 2 Kaplan Heart Center, Kaplan Medical Center, Faculty of Medicine, Hebrew University of Jerusalem, Pasternak St. P.O. Box 1, Rehovot 76100, Israel; haberdan@gmail.com (D.H.); sarah_s2@clalit.org.il (S.S.); ortalhagege@gmail.com (O.T.); 3 Heart Institute, Hadassah-Hebrew University Medical Center, Jerusalem 90000, Israel; mehseti.ibrahimli@gmail.com (M.I.); ivailortonchev@gmail.com (I.T.); alonaperetz@gmail.com (A.P.); ronenbe@ekmd.huji.ac.il (R.B.); 4 Heart Institute, Wolfson Medical Center, Ha-Lokhamim St. 62, Holon 5822012, Israel; danen040@gmail.com

**Keywords:** mitral regurgitation, acute decompensated heart failure, percutaneous mitral valve intervention

## Abstract

The role of percutaneous mitral valve repair (PMVr) in management of high-risk patients with severe mitral regurgitation (MR) and acute decompensated heart failure (ADHF) is undetermined. We screened all patients who underwent PMVr between October 2015 and March 2020. We evaluated immediate, 30-day, and 1-year outcomes in patients who underwent PMVr during hospitalization due to ADHF as compared to elective patients. From a cohort of 237 patients, we identified 46 patients (19.4%) with severe MR of either functional or degenerative etiology who underwent PMVr during index hospitalization due to ADHF, including 17 (37%) critically ill patients. Patients’ mean age was 75.2 ± 9.8 years, 56% were males. There were no differences in background history between ADHF and elective patients. Patients with ADHF were at higher risk for surgery, reflected in higher mean EuroSCORE II, compared with elective patients. After PMVr, we observed higher 30-day mortality rate in ADHF patients as compared to the elective group (10.9% vs. 3.1%, respectively, *p* = 0.042). One-year mortality rate was similar between the groups (21.7% vs. 17.9%, *p* = 0.493). Clinical and echocardiographic follow-up showed improvement of NYHA functional class and sPAP reduction in both groups ((54 ± 15 mmHg to 50 ±15 in the elective group (*p* = 0.02), 58 ± 13 mmHg to 52 ± 12 in the ADHF group (*p* = 0.02)). PMVr could be an alternative option for treatment of patients with severe MR and ADHF.

## 1. Introduction

Acute decompensated heart failure (ADHF) is associated with high rates of mortality and morbidity including frequent short-term unplanned hospitalizations [1,2]. The management of ADHF remains challenging. It is important to identify and treat specific precipitation factors that lead to ADHF. The most common precipitants are noncompliance with medications or dietary restrictions, uncontrolled hypertension, ischemia, arrhythmias, and exacerbation of chronic obstructive pulmonary disease [3].

Mitral regurgitation (MR) is the most common valvular heart disorder and a large cause of heart failure (HF) exacerbation [4,5]. In advanced HF, MR increases the burden of the failing ventricle and decreases effective stroke volume [6]. Both primary (degenerative) and secondary (functional) MR may lead to ADHF. The underling mechanism of degenerative MR in ADHF is disruption of any component of the apparatus or surrounding anatomy that can lead to MR (such as flail leaflet due to cord rupture) [4]. Functional MR is a ventricular disease that occurs despite a structurally normal mitral valve, which can be classified as having an ischemic, or non-ischemic etiology, the former being the most common and occurring after myocardial infarction (MI). The mechanism of acute ischemic MR complicating an acute MI includes the rupture of a papillary muscle or acute left ventricle (LV) remodeling, which leads to apical and posterior displacement of the papillary muscles, causing leaflet tethering and reduced closing forces [7,8]. The pre-dominant mechanism for non-ischemic functional MR is an increased effective regurgitant orifice from annular dilation and loss of annular contraction. It can be caused by idiopathic dilated cardiomyopathy (DCM), long-standing hypertension, and myocarditis [9]. Management of patients with ADHF and severe MR is often challenging.

The management of ADHF during index hospitalization is often different from that of chronic HF as in-patient treatment consists primarily of hemodynamic stabilization, symptom relief, and prevention of short-term morbidity and mortality [4]. The role of Percutaneous Mitral Valve repair (PMVr) is less known in the management of high-risk patients with ADHF and severe MR during index hospitalization. In our study, we evaluated the immediate, 30-day, and 1-year outcomes of PMVr in patients with ADHF and severe MR of both functional and degenerative etiology without adequate response to the optimal medical therapy.

## 2. Materials and Methods

### 2.1. Study Population

We screened all patients who underwent Transcatheter Edge-to-Edge Repair (TEER) with MitraClip^®^ implantation at two medical centers (Hadassah Medical Center and Kaplan Medical Center) between October 2015 and March 2020. All patients received thorough verbal and written explanations about MitraClip implantation procedure and signed informed consents. The institutional committee for human studies of both centers approved the study protocol. We evaluated patients with severe MR who were hospitalized due to ADHF or electively, including a cohort of critically ill patients with refractory HF. Patients with ADHF presented with a rapid onset and worsening of symptoms of HF and New York Heart Association (NYHA) functional class above III, often in the context of pre-existing cardiomyopathy. The diagnosis of ADHF was based on clinical features that included pulmonary congestion and dependence on intravenous (IV) continuous infusions of diuretics (high dose of Furosemide). Critically ill patients with cardiogenic shock presented with low systolic blood pressure (below 90 mmHg), clinical signs of poor tissue perfusion (oligo/anuria, cyanosis, cool extremities, and altered mentation), and were dependent on inotropic/vasopressors drugs (Norepinephrine or Dopamine in inotropic dose) in order to increase blood pressure, and/or Intra-Aortic Balloon Pump (IABP) support. In our study, the IABP was placed only in post-MI patients with acute ischemic MR. Elective patients presented with stable HF symptoms (shortness of breath during regular or slight exertions) and scheduled for a planned procedure. The Heart Team that included HF cardiologist, Interventional cardiologist, Cardiac surgeon, and Interventional echocardiographer evaluated each case. TEER was performed in high-risk-for-surgery, symptomatic patients with moderate-to-severe or severe functional mitral regurgitation (MR) who remained symptomatic despite stable doses of maximally tolerated Guideline-Directed Medical Therapy (GDMT) plus cardiac resynchronization therapy, if appropriate, or for the treatment of significant symptomatic degenerative MR according to an FDA-approved indication.

### 2.2. Echocardiographic Assessment

Transthoracic (TTE) and transesophageal echocardiography (TEE) were performed to evaluate the severity of MR and to assess suitability for MitraClip^®^ implantation. We used an available ultrasound diagnostic systems (Vivid 7 and Vivid E9, GE Medical Systems, Milwaukee, WI, USA and Philips IE 33, Royal Philips Electronics, Amsterdam, The Netherlands) with two-dimensional imaging and Doppler data including pulsed wave and continuous wave (CW) imaging. The severity of MR was initially assessed by integrated multiparametric visual evaluation tool in accordance with standard clinical practice (incorporating 2D, spectral and color Doppler images) using an ordinal scale (grading 0—no MR, 1+ mild MR, 2+ moderate MR, 3+ moderate to severe MR, 4+ severe MR), and effective regurgitant orifice area (EROA) was calculated based on proximal isovelocity surface area (PISA) method with 40 mm^2^ as cutoff for severe MR [10]. The etiology of MR was evaluated according to the European Association of Echocardiography recommendations [11]. We evaluated patients with severe degenerative MR due to flail leaflet and ruptured cord as well as severe functional MR due to ischemic (after a recent MI) and non-ischemic etiology (secondary to DCM). Functional MR was defined in the case of abnormal function of normal leaflets in the context of impaired LV function, without echocardiographic signs of other intrinsic pathology of the leaflets, chordae, and papillary muscles [4]. Pulmonary artery systolic pressures (sPAP) were estimated by calculating maximal tricuspid pressure gradient, using CW Doppler, and the right atrial pressure based on inferior vena cava size and changes during inspiration. A sPAP ≤ 40 mmHg was considered normal [11].

### 2.3. Intervention

Transcatheter edge-to-edge PMVr was performed by implantation of device “MitraClip^®^” (Abbott, Abbott Park, IL, USA)—a catheter-based system, which consists of a steerable 24 Fr guide catheter and a clip delivery system. The clip device system was delivered to the left atrium via a trans-septal puncture, then advanced into the LV and retracted grasping the mitral valve leaflets. The second or third clip device was placed at operator discretion to obtain additional MR reduction. All clips were implanted under general anesthesia and fluoroscopic and TEE guidance [12]. Procedural success was defined as MR reduction to grade 1+ or 2+ after implantation of at least one clip. All patients were transferred to our intensive care cardiac unit (ICCU) after the procedure for further observation.

### 2.4. Outcomes

We evaluated clinical outcomes including NYHA functional class, 30-day and 1-year mortality, and 1-year rehospitalization after PMVr in patients with severe MR admitted electively or due to ADHF. Additional outcomes included hemodynamic stabilization, echocardiographic assessment of MR, LV end-diastolic diameter (LVEDD), and sPAP after PMVr. For the follow-up assessment, all patients were scheduled for 30-day and 12-months as outpatients or telephone visits.

### 2.5. Statistical Analysis

Our analyses were performed using the entire cohort, comparing between patients presenting with ADHF or electively admitted. Patient characteristics are reported according to variable properties. Continuous variables were expressed as mean ± standard deviation or median and interquartile range, where appropriate. Those with a normal distribution are reported as mean (± standard deviation), and differences between subgroups were tested using the student’s *t*-test. Those without a normal distribution are reported (interquartile range), and differences between subgroups were tested using the Mann–Whitney U. Categorical variables were presented as counts and percentages.

Follow-up time was calculated using Kaplan–Meier estimate of potential follow-up. Kaplan–Meier curves with the log-rank test were used to compare survival. All clinical events were analyzed by time to first event for Kaplan–Meier analysis. A two-tailed *p*-value ≤ 0.05 is regarded as statistically significant. The IBM Statistical Package for the Social Sciences (SPSS) Statistics 26.0 (IBM Corp., Armonk, NY, USA) was utilized to perform the analyses.

## 3. Results 

From a cohort of 237 patients who underwent PMVr implantation in two centers, we identified 46 patients (19.4%) with ADHF. The other 191 patients (81%) were admitted electively. The mean patient age was 75 ± 10 year, 40% were females.

Baseline clinical characteristics of study population before PMVr are shown in Table 1.

There were no differences in age and sex between ADHF and elective patients. There was no significant difference in background illnesses: Diabetes mellitus, Hypertension, Hyperlipidemia, history of smoking, and previous stroke between the two groups. There was a tendency toward higher prevalence of previous MI in the ADHF group compared with the elective group (45.7% vs. 36.1%, *p* = 0.24). Patients presented with ADHF had significantly higher prevalence of HF-related hospitalizations during the last year as compared with elective patients (95.7% vs. 80.0%, *p* = 0.01). There were no significant differences in medical treatment between the two groups, except for lower use of beta-blockers and mineralocorticoid-receptor antagonists (MRA) in the ADHF group compared to the elective group [66.7 vs. 87% (*p* = 0.02) and 33.3 vs. 52.3% (*p* = 0.02), respectively]. Patients with ADHF were at higher risk for surgery reflected in higher mean EuroSCORE II, compared with elective patients (15.9 ± 13.1% vs. 9.0 ± 8.5% respectively, *p* = 0.01). The prevalence of advanced renal failure with GFR < 30 mL/min was lower in the ADHF group as compared with elective patients (11% vs. 26%, *p* = 0.04). ADHF patients were more likely to present with advanced HF and higher grade of NYHA functional class IV (84.4% vs. 31.2%, *p* = 0.01) as compared to elective patients.

Out of 46 ADHF patients, 17 patients (37.0%) were critically ill. All critically ill patients were dependent on IV infusions of inotropes/vasopressors prior to the PMVr in order to increase blood pressure despite MR exacerbation. Fifteen patients (32.6%) with post-MI acute functional MR depended on IABP for mechanical support before the PMVr.

Baseline echocardiographic characteristic are shown in Table 2.

**Figure 1:**
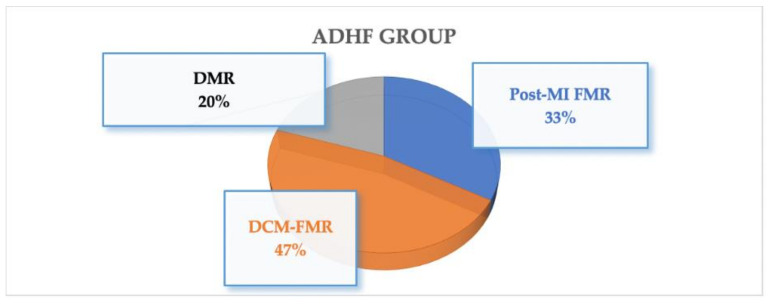
Etiology of MR in patients admitted due to ADHF. MR = mitral regurgitation; ADHF = acute decompensated heart failure; MI = myocardial infarction; DMR = degenerative mitral regurgitation; FMR = functional mitral regurgitation; DCM = dilated cardiomyopathy.

On average, patients in the ADHF group had higher sPAP compared with elective patients (58 ± 14 mmHg vs. 53 ± 15 mmHg, *p* = 0.06). Among patients admitted due to ADHF, 15 cases (32.6%) had functional ischemic MR due to a recent MI. The other 22 patients (47.2%) had functional MR due to decompensated DCM. Nine patients (19.6%) had severe degenerative MR secondary to ruptured cord (Figure 1).

Reduction of MR severity was achieved in 209 out of 237 patients with device success of 89.9%. Patients presented with ADHF were implanted with more clips compared with the elective group (16%, 64%, and 20% vs. 36%, 55%, and 9% for one, two, and three clips, respectively, *p* = 0.01). Peri-procedural complications were observed in 14 patients (4.6%). There were no differences in procedural success and periprocedural complications between the two groups (Table 3).

Post-procedural echocardiography showed marked MR reduction in patients of both ADHF and elective admission groups. There was a significant mean left atrial V-wave reduction from 33 ± 13 mmHg to 18 ± 8mmHg after the procedure (*p* < 0.01). V-wave reduction was significant in both groups. There were no signs of iatrogenic significant mitral stenosis with mean MV gradient of 4.1 ± 2.0 mmHg. MV gradient was higher in the non-ADHF presentation group (4.3 ± 2.1 mmHg vs. 3.4 ± 1.4 mmHg, *p* = 0.01) (Table 4).

The median clinical follow-up period was 620 days (interquartile range of 262–888 days).

There was an improvement in the clinical status of patients in the ADHF group during the immediate post-PMVr period. Continuous infusion of diuretics was discontinued in 41 out of 46 ADHF patients (89.1%). Among critically ill patients, 14 out of 17 patients (82.4%) were weaned from IV medications and 13 out of 15 patients (86.7%) were successfully weaned from IABP mechanical support. There was a significantly higher rate of in-hospital death in the ADHF group as compared with elective patients (10.9% vs. 2.6%, *p* = 0.026) (Table 5).

After PMVr, there was an improvement of NYHA functional class in both groups of patients. There was no statistically significant changes of LVEDD in both groups at the follow-up period. There was a significant reduction of sPAP in both group of patients during follow-up: 54 ± 15 mmHg to 50 ± 15 in the non-ADHF group (*p* = 0.02) and 58 ± 13 mmHg to 52 ± 12 in the ADHF group (*p* = 0.02) (Table 6).

**Figure 2:**
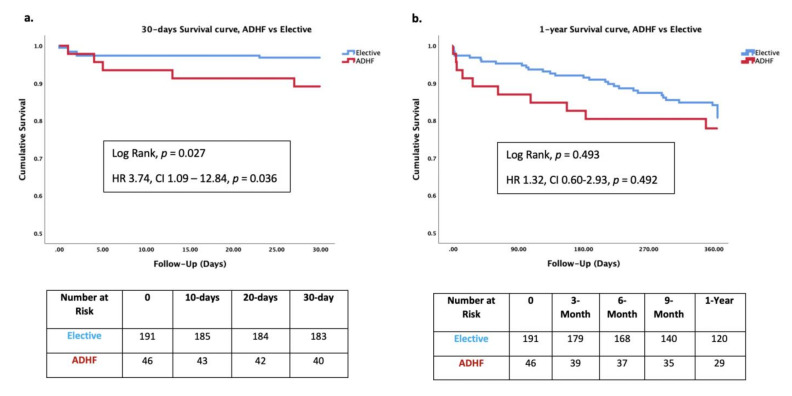
Kaplan–Meier survival analysis of 237 patients who underwent PMVr for severe MR between 2015 and 2020 by the ADHF group and elective patients. *p*-value for log rank test. (**a**). 30-day analysis. **(b**). One-year analysis. PMVr = percutaneous mitral valve repair; ADHF = acute decompensated heart failure; MR = mitral regurgitation.

Thirty-day mortality rate was higher for patients presenting with ADHF compared to patients with elective admission (10.9% vs. 3.1%, *p* = 0.042). One-year mortality rate was similar in both groups of patients (21.7% vs. 17.9%, *p* = 0.4) (Table 5, Figure 2).

**Figure 3:**
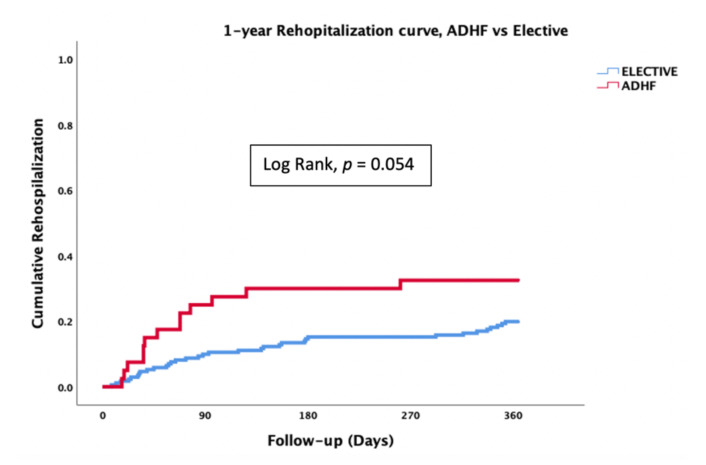
Kaplan–Meier analysis for 1-year HF-related rehospitalizations of 237 patients who underwent PMVr for severe MR between 2015 and 2020 by the ADHF group and elective patients (*p* = 0.054). ADHF = acute decompensated heart failure.

In addition, there was a tendency toward a higher 1-year HF rehospitalizations rate in the ADHF group as compared with elective patients (33% vs. 20%) (Table 5, Figure 3).

**Figure 4:**
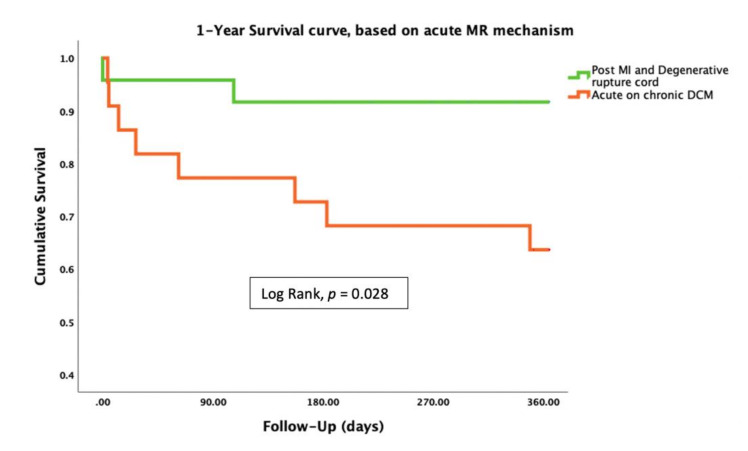
Kaplan–Meier survival analysis of 46 ADHF patients who underwent PMVr for severe MR between 2015 and 2020 by MR etiology. *p*-value for log rank test. PMVr = percutaneous mitral valve repair; ADHF = acute decompensated heart failure; MR = mitral regurgitation; MI = myocardial infarction; DMR = degenerative mitral regurgitation; DCM = dilated cardiomyopathy.

Among the ADHF group, patients with acute presentation (degenerative rupture cord or acute MI) had better prognosis as compared to the acute-on-chronic DCM group (Figure 4).

Multivariable Cox regression analysis demonstrated that EuroSCORE II and sPAP at baseline were independent predictors of increased mortality at 30 days (Table 7). The only significant parameter predicting 1-year mortality was EuroSCORE II (HR 1.03, CI 1–1.05, *p* = 0.05) (Table S1). Importantly, EuroSCORE II was higher among patients with acute presentation and might impact their outcomes.

## 4. Discussion

Advances in both surgical and catheter-based therapies resulted in the extension of interventional treatments to the older, sicker population of patients with MR [4].

Recent data suggests that PMVr may be considered for elective high-risk patients with severe symptomatic functional and degenerative MR and HF as an alternative to surgical valve repair [4,12,13,14].

The role of PMVr is less known in the management of high-risk patients with ADHF and severe MR. In our study, we evaluated the impact of PMVr on outcomes of patients with severe MR admitted due to ADHF that required urgent PMVr and compared them with patients who underwent elective PMVr. Although short-term mortality was higher among ADHF population, 1-year mortality was similar between the groups.

In addition, we showed that PMVr during the index hospitalization is safe and effective in high-risk ADHF patients with good procedural success. It is interesting to note that in the ADHF group, despite receiving more clips, the MV gradients were lower. We might suggest that these patients have dilated ventricles with more dilated annulus. Therefore, the MV gradients were lower even with more clips implanted as compared with elective patients.

Previously, we showed that the PMVr could be a “bail-out” option in treatment of critically ill patients with refractory HF and severe functional MR [15]. In the current study, PMVr allowed to stabilize patients with ADHF and functional MR after recent MI as well as patients with acute degenerative MR who presented with ruptured cord.

Several studies evaluated the impact of MR severity, etiology, and persistency on outcomes of patients with ADHF.

The recent ARIC study showed that the higher MR severity was associated with worse prognosis in patients ADHF with excess 1-year mortality. However, a limitation in this study is the lack of differentiation between the underlying etiology of MR (primary vs. secondary/functional), though the authors suggest that a substantial portion of MR must have been functional [16].

Kubo et al. showed that in patients hospitalized for ADHF, dynamic severe MR on hospital arrival was associated with poorer outcomes than non-significant MR and had similar risk to persistent severe MR. The risk of dynamic MR was consistent in the subgroups of patients with reduced (<45%) and preserved LV ejection fraction [17].

In our study, we evaluated patients with severe MR of either functional or degenerative etiology. We showed that the ADHF group of patients presented with acute MR (degenerative ruptured cord or post-MI) had better prognosis as compared to patients with acute-on-chronic DCM.

Impact of acute ischemic functional MR on prognosis of patients with acute MI was evaluated by Estevez-Loureiro (EREMMI registry) [18] and by Haberman et al. [19,20], which showed immediate reduction in left atrial V-wave, pulmonary artery pressure, improvement in NYHA functional class, and improved hemodynamic parameters after PMVr with MitraClip implantation.

Tomás Benito-González et al. examined procedural and clinical outcomes among 85 patients in the single center population undergoing PMVr within an admission for ADHF. In line with our findings, they found MR reduction and NYHA functional class improvement with no differences in 1-year mortality between urgent or elective PMVr groups. Interestingly, contrary to our findings, 30-day mortality was also similar between the groups; however, that might be related to a small cohort, which was underpowered to detect the survival differences [21].

Recently, Richard G Jung et al. conducted multicenter analysis and showed that PMVr may improve short- and intermediate-term mortality in high-risk patients with cardiogenic shock and moderate-to-severe MR. Patients presented with cardiogenic shock and functional MR benefit from early intervention with TEER [22]. In addition, Haberman D. et al. showed that early intervention with TEER might mitigate the poor prognosis associated with conservative therapy in patients with post-MI MR. PMVr can serve as an alternative for surgery in reducing MR for high-risk patients [23].

The limitations of our study include its observational retrospective nature in a two-center population. The groups were not randomized into the different treatment arms. Additional clinical (such as NT-Pro-BNP), echocardiographic data (such as EROA) was not fully available. There was non-adherence to GDMT even in a large fraction of elective patients. In our study, critically ill patients were treated with inotropic dose of dopamine to increase blood pressure as well as IABP for mechanical support, which is not a recommended therapy for cardiogenic shock. According to recent guidelines, routine IABP use in cardiogenic shock is contraindicated, except in cases of mechanical MI complications such as acute mitral regurgitation or ventricular septal defect. In our study, the IABP was placed only in post-MI patients with acute ischemic functional MR. Large, multicenter and randomized trials are needed to confirm this data and eliminate the selection bias.

## 5. Conclusions

PMVr using MitraClip^®^ therapy could be an alternative option for treatment of patients presented with ADHF and severe MR of both degenerative and functional etiology.

## Figures and Tables

**Table 1:** Baseline clinical characteristics of study population before PMVr.

Clinical Characteristics	Patient Population			*p*-Value
	Total Population, *n* = 237	Patients with ADHF, *n* = 46	Elective Patients, *n* = 191	
Age, years	75.2 ± 9.8	73.0 ± 9.4	75.9 ± 9.8	0.08
Male, *n* (%)	139 (56)	25 (54)	114 (60)	0.51
Known CAD, *n* (%)	146 (62)	31 (67)	115 (60)	0.50
Post MI, *n* (%)	90 (38)	21(46)	69 (36)	0.24
Hypertension, *n* (%)	204 (86)	40 (86)	164 (87)	1.00
Diabetes Mellitus, *n* (%)	91 (38)	21 (45)	70 (36)	0.21
Hyperlipidemia, *n* (%)	188 (79)	35 (76)	153 (80)	0.55
Smoker, *n* (%)	88 (37)	18 (39)	70 (37)	0.41
S/P CVA, *n* (%)	44 (19)	8 (17)	36 (19)	1.00
AFIB, *n* (%)	113 (48)	23 (51)	90 (47)	0.34
Post-thoracotomy, *n* (%)	65 (27)	6 (13)	59 (31)	0.03
HF-related hospitalizations last year, %	197 (83)	44 (96)	153 (80)	>0.01
Cardiac-related drug therapy:				
ACE-inhibitors or ARBs, *n* (%)	169 (71)	32 (69)	137 (72)	0.17
Beta blockers, *n* (%)	201 (84)	34 (73)	167 (88)	0.02
MRA, *n* (%)	109 (46)	15 (33)	100 (52)	0.02
Furosemide, *n* (%)	206 (87)	38 (83)	168 (88)	0.34
EuroSCORE II	10.3 ± 10.8	15.9 ±13.1	9.0 ± 8.5	0.01
Hb, g/L	11.8 ± 1.6	12.0 ± 1.7	11.7 ± 1.6	0.46
Cr, mg/dL	138 ± 85	111.8 ± 41.8	146.2 ± 93.3	0.1
GFR, mL/min	53 ± 24	57.9 ± 19.4	51.2 ± 25.4	0.27
GFR < 30 mL/min, *n* (%)	54 (23)	5 (11)	49 (26)	0.04
Mean NYHA functional class	3.4 ± 0.6	3.7 ± 0.8	3.3 ± 0.5	0.01
NYHA functional class IV, %	101 (43)	41 (89)	60 (32)	0.01

ADHF = acute decompensated heart failure; CAD = coronary artery disease; MI = myocardial infarction; CVA = cerebrovascular accident; AFIB = atrial fibrillation; HF = heart failure; ACE = Angiotensin-converting enzyme; ARB = Angiotensin II receptor blockers; MRA = mineralocorticoid receptor antagonist; Hb = hemoglobin; Cr = creatinine; GFR = Glomerular filtration rate; NYHA = New York Heart Association.

**Table 2:** Baseline echocardiographic characteristics of patient population before PMVr.

Echocardiographic Characteristics	Patient Population			*p*-Value
	Total Population, *n* = 237	ADHF Patients, *n* = 46	Elective Patients, *n* = 191	
MR grade 3^+^, *n* (%)	55 (23)	8 (18)	47 (24)	0.64
MR grade 4^+^, *n* (%)	182 (77)	38 (82)	144 (76)	
EROA, mm^2^ (SD)	41 ± 11	43 ± 11	41 ± 10	0.81
LVEF, % (SD)	35.5 ± 13.4	32.4 ± 13.6	36.2 ± 13.3	0.15
<40%, *n* (%)	159 (67)	37 (79)	122 (64)	0.23
40–50%, *n* (%)	34 (14)	4 (9)	30 (16)	
>50%, *n* (%)	44 (19)	5 (12)	41 (20)	
LVEDD, mm (SD)	57.4 ± 10.2	58.5 ± 11.2	57.2 ± 9.9	0.57
sPAP, mmHg (SD)	54.5 ± 15.0	58.4 ± 13.7	53.5 ± 15.2	0.07

PMVr = percutaneous mitral valve repair; ADHF = acute decompensated heart failure; EROA = effective regurgitant orifice area; MR = mitral regurgitation; LVEF = left ventricle ejection fraction; LVEDD = left ventricle end-diastolic diameter; sPAP = systolic pulmonary artery pressure.

**Table 3:** Periprocedural characteristics of patient population underwent PMVr.

Characteristic	Total Population, *n* = 237	ADHF Patients, *n* = 46	Elective Patients, *n* = 191	*p*-Value
Procedural success	213/237 89.9%	44/46 91.3%	169/191 89.3%	0.43
One clip implantation, %	76/237 32%	7/46 16%	69/191 36%	0.01
Two-clip implantation, %	135/237 57%	30/46 64%	105/191 55%	
Three-clip implantation, %	26/237 11%	9/46 20%	17/191 9%	
Procedural Complications	14/237 4.6%	2/46 4.3%	12/191 6.3%	0.47

PMVr = percutaneous mitral valve repair; ADHF = acute decompensated heart failure.

**Table 4:** Hemodynamic and echocardiographic characteristics of patient population with severe MR before and immediately after PMVr.

	Total Population, *n* = 237	ADHF Patients, *n* = 46				Elective Patients, *n* = 191			
Variable	Pre	Pre	Post	Follow-up	*p*-value	Pre	Post	Follow-up	*p*-value
MR (1–4)	3.72 ± 0.53	3.72 ± 0.59	1.43 ± 0.69	N/A	< 0.01	3.71 ± 0.54	1.59 ± 0.78	N/A	<0.01
V-wave, mmHg	32.8 ± 13.4	32.1 ± 13.6	17.9 ± 8.0	N/A	<0.01	36.5 ± 12.4	19.8 ± 7.5	N/A	<0.01
MV gradient, mmHg	N/A	N/A	3.38 ± 1.41	N/A		N/A	4.25 ± 2.11	N/A	

PMVr = percutaneous mitral valve repair; ADHF = acute decompensated heart failure; MR = mitral regurgitation; MV = mitral valve; Pre = pre-procedure; Post = post-procedure.

**Table 5:** Mortality and 1-year rehospitalizations rate of patient population that underwent PMVr.

Mortality Rate	Total Population, *n* = 237	ADHF Patients, *n* = 46	Elective Patients, *n* = 191	*p*-Value
In-hospital mortality, *n* (%)	10/237 4.2%	5/46 10.9%	5/191 2.6%	0.026
30-day mortality, *n* (%)	11/237 4.6%	5/46 10.9%	6/191 3.1%	0.042
1-year mortality, *n* (%)	44/237 18.6%	10/46 21.7%	34/191 17.9%	0.491
1-year HF-rehospitalizations, *n* (%)	53/237 22.3%	15/46 33%	38/191 20%	0.09

PMVr = percutaneous mitral valve repair; ADHF = acute decompensated heart failure.

**Table 6:** Clinical and echocardiographic outcomes of patient population with severe MR underwent PMVr.

Variable	Total Population, *n* = 237	ADHF Patients, *n* = 46			Elective Patients, *n* = 191		
	Pre	Pre	Follow-Up	*p*-Value	Pre	Follow-Up	*p*-Value
NYHA functional class (0–4)	3.31 ± 0.49	3.93 ± 0.26	2.27 ± 0.59	<0.01	3.31 ± 0.49	2.21 ± 0.54	<0.01
LVEDD, mm	57.2 ± 10.0	59.5 ± 11.1	55.6 ± 7.9	0.22	57.2 ± 10.0	57.4 ± 10.9	0.86
sPAP, mmHg	55.5 ± 14.7	58.2 ± 13.5	51.9 ± 12.1	0.02	54.1 ± 15.0	50.1 ± 15.1	0.02

PMVr = percutaneous mitral valve repair; ADHF = acute decompensated heart failure; MR = mitral regurgitation; MV = mitral valve; Pre = pre-procedure; NYHA = New York Heart Association; LVEDD = left ventricle end-diastolic diameter; sPAP = systolic pulmonary artery pressure.

**Table 7:** Logistic regression analysis for prediction of cardiovascular mortality during 30-day follow-up after PMVr.

Variable	Univariable			Multivariable		
	RR	CI	*p*-Value	RR	CI	*p*-Value
Acute Clip	3.74	1.01–12.84	0.04	4.85	0.4–55.1	0.2
Age	1.01	0.94–1.07	0.95			
Gender	1.21	0.36–4.07	0.76			
BMI	1.06	0.97–1.16	0.22			
Diabetes Mellitus	1.05	0.29–3.82	0.94			
Hyperlipidemia	1.20	0.25–5.73	0.82			
Smoker	1.14	0.52–2.50	0.75			
COPD	1.17	0.14–9.71	0.89			
CAD	2.10	0.55–8.03	0.28			
S/P MI	1.67	0.43–6.48	0.43			
CABG	1.34	0.32–5.51	0.69			
EuroSCORE II	1.05	1.01–1.09	0.02	1.08	1.02–1.14	<0.01
Base NYHA FC 4	2.95	0.72–12.1	0.1	1.6	0.11–21.9	0.72
Mechanism (DMR)	0.81	0.20–3.32	0.77			
LVEF (Grade)	0.81	0.51–1.28	0.37			
sPAP	1.05	1.0–1.1	0.05	1.06	1.01–1.12	0.02

PMVr = percutaneous mitral valve repair; MI = myocardial infarction; BMI = body mass index; COPD = chronic obstructive pulmonary disease; CAD = coronary artery disease; NYHA FC = New York Heart Association functional class; CABG = coronary artery bypass graft; DMR = degenerative mitral regurgitation; LVEF = left ventricle ejection fraction; sPAP = systolic pulmonary artery pressure.

## Data Availability

The data that supports the findings of this study are available from the corresponding author, M.S., upon reasonable request.

## Supplementary Materials

The following are available online at https://www.mdpi.com/article/10.3390/jcm10245849/s1, Table S1: Logistic regression analysis for prediction of cardiovascular mortality during 1-year follow-up after PMVr.
