# Supplementary material for: Percutaneous Mitral Valve Repair in Patients with Severe Mitral Regurgitation and Acute Decompensated Heart Failure
Source: J Clin Med. 2021 Dec 13;10(24):5849. doi: 10.3390/jcm10245849 (PMC8704045; doi:10.3390/jcm10245849)
Supplement: Supplementary file 1 [file jcm-10-05849-s001.zip › jcm-1403896-supplementary.pdf]

Journal of
Clinical Medicine

MDPI

Supplementary Materials

Supplementary Table S1. Logistic regression analysis for prediction of cardiovascular mortality during 1-year follow-up after PMVr.

Variable	Univariable			Multivariable		
	**RR**	**CI**	***p*-value**	**RR**	**CI**	***p*-value**
Acute Clip	1.32	0.60–2.93	0.49			
Age	1.02	0.98–1.06	0.30			
Gender	1.17	0.60–2.27	0.65			
BMI	1.04	0.99–1.10	0.16			
Diabetes Mellitus	1.29	0.63–2.61	0.49			
Hyperlipidemia	1.04	0.46–2.36	0.91			
COPD	1.02	0.36–2.864	0.96			
CAD	1.08	0.54–2.17	0.83			
S/P MI	1.13	0.56–2.26	0.73			
CABG	1.92	0.95–3.90	**0.07**			
EuroSCORE II	1.03	1.00–1.05	**0.05**			
Base NYHA FC 4	1.13	0.57–2.25	0.73			
Mechanism (DMR)	0.77	0.38–1.56	0.77			
LVEF (Grade)	0.92	0.40–2.12	0.84			
sPAP	1.01	0.99–1.04	0.39			

PMVr = percutaneous mitral valve repair; MI = myocardial infarction; BMI = body mass index; COPD = chronic obstructive pulmonary disease; CAD = coronary artery disease; NYHA FC = New York Heart Association functional class; CABG = coronary artery bypass graft; DMR = degenerative mitral regurgitation; LVEF = left ventricle ejection fraction; sPAP = systolic pulmonary artery pressure.
